# Psychological distress differentiates subjective health within a vulnerable subgroup of patients with immune-mediated inflammatory diseases

**DOI:** 10.3389/fpsyg.2026.1884317

**Published:** 2026-08-31

**Authors:** Tessa S. Folkertsma, Eline J. Mertens, Damien S. E. Broekharst, Robert M. Vodegel, Greetje J. Tack, Maya J. Schroevers, Sjaak Bloem, Reinhard Bos

**Affiliations:** 1 Department of Dermatology, Frisius MC, Leeuwarden, Netherlands; 2 Department of Gastroenterology, Frisius MC, Leeuwarden, Netherlands; 3 Department of Rheumatology, Frisius MC, Leeuwarden, Netherlands; 4 Department of Health Psychology, University Medical Center Groningen, Groningen, Netherlands; 5 Center for Marketing and Supply Chain Management, Nyenrode Business University, Breukelen, Netherlands

**Keywords:** health psychology, immune-mediated inflammatory diseases, integrated care, patient stratification, personalized supportive care, psychological distress, subjective health experience model

## Abstract

**Background:**

Patients with immune-mediated inflammatory diseases (IMIDs) who score low on both acceptance and perceived control are classified as segment 4 in the Subjective Health Experience (SHE) model. They represent the most psychologically vulnerable group. Prior work in oncology has identified heterogeneity within this segment, but whether similar differentiation exists in IMIDs remains unknown. Therefore, the present study examines heterogeneity within SHE segment 4 in IMIDs by comparing moderate and severe subgroups on psychological and disease-related outcomes.

**Methods:**

A cross-sectional survey was conducted among 500 Dutch adults with physician-confirmed IMIDs. Of these, 166 were classified in segment 4 and subdivided into moderate (*n* = 96) and severe (*n* = 70) subgroups based on median acceptance and control scores. Group differences in psychological (i.e., subjective health, Health-Related Quality of Life (HRQoL), psychological distress) and disease-related outcomes (i.e., self-reported disease severity and activity) were assessed using *t*-tests and chi-square analyses. Multiple linear regression analyses were performed to determine whether subgroup differences remained after adjustment for demographic and clinical characteristics.

**Results:**

The severe subgroup reported a significantly poorer subjective health, lower HRQoL, and higher levels of anxiety, depression, and overall psychological distress, compared with the moderate subgroup. These associations remained significant after adjustment for age, gender, education, diagnosis, and comorbidity burden. No significant differences were observed in disease activity, disease impact, or comorbidity burden. Exploratory diagnosis-specific analyses suggested that subgroup differences were present across diagnoses, although subgroup sizes were limited.

**Conclusion:**

Meaningful psychological heterogeneity exists within SHE segment 4 in IMIDs and appears to be primarily related to psychological distress and quality of life rather than disease-related factors. These findings support the potential value of the SHE model for identifying patients who may benefit from personalized supportive care.

## 1 Introduction

Chronic immune-mediated inflammatory diseases (IMIDs), such as rheumatoid arthritis (RA), psoriasis (PsO), inflammatory bowel disease (IBD), and axial spondyloarthritis (axSpA), affect millions globally and constitute an increasing health burden (Gelabert-Mora et al., 2024). IMIDs are characterized by persistent inflammation, fluctuating disease activity, and long-term symptom burden that impacts various life domains (McInnes and Gravallese, 2021). In addition to pain, fatigue, and functional limitations, these conditions affect emotional wellbeing, social participation, and work productivity (James et al., 2024; Spierings et al., 2020). Despite significant advances in biomedical treatment targeting inflammation, many patients continue to experience substantial limitations in daily functioning and quality of life (James et al., 2024; Spierings et al., 2020).

Although biomedical management primarily targets inflammatory processes, objective disease activity markers explain only part of the variability in how patients experience their health. Clinical markers often show only moderate correlations with patient-reported outcomes. Moreover, substantial levels of depression and anxiety are documented across IMIDs (Bacconnier et al., 2015; Tarar et al., 2022), and patients with comparable clinical severity frequently report substantially different levels of Health-related quality of life (HRQoL) and psychological wellbeing (Matcham et al., 2013; Matcham et al., 2016; Mikocka-Walus et al., 2016). These discrepancies suggest that biomedical indicators alone do not fully explain lived experience in IMIDs. Psychological determinants may also play a prominent role, reflecting the close interplay between psychological functioning and inflammatory activity (Matcham et al., 2016; Mikocka-Walus et al., 2016; Peppas et al., 2021; Tarar et al., 2022).

Psychological distress is consistently elevated across IMIDs and is strongly associated with impaired subjective health. Across RA, IBD, PsO, and axSpA, elevated rates of depression and anxiety are well-documented, with prevalence estimates ranging from 20–40% depending on the condition and measurement instrument used (Dalgard et al., 2015; Dowlatshahi et al., 2014; Knowles et al., 2018; Matcham et al., 2013; Mikocka-Walus et al., 2016; Reddy et al., 2022; Zhao et al., 2018). Depression in particular has been associated with higher disease activity, worse clinical outcomes (Rathbun et al., 2013), and demonstrates substantial heterogeneity in HRQoL even among patients with comparable clinical severity (Song et al., 2024). Across these conditions, psychological distress frequently persists during periods of low inflammatory activity or remission (Knowles et al., 2018; Mikocka-Walus et al., 2016), suggesting that variation in experienced health extends beyond objective disease activity.

In addition to psychological distress, multimorbidity is common in individuals with IMIDs and is associated with worse health-related quality of life, increased healthcare utilization, and greater clinical complexity (Barnett et al., 2012; Valderas et al., 2009). Patients with multiple chronic conditions show more rapid declines in physical and mental functioning than those with a single condition (Bayliss et al., 2005). However, the number of comorbidities explains only part of the variability in subjective wellbeing (Fortin et al., 2006; Nicholson et al., 2019). This suggests that patients with a comparable disease burden may nevertheless experience and adapt to their condition differently. Indeed, research indicates that psychological adaptation, disease perceptions, and coping mechanisms influence how disease burden translates into patients’ overall health experience (Helgeson and Zajdel, 2017; Leventhal et al., 1980).

Several psychological models have sought to explain this variability in adaptation, including frameworks centered on stress, self-regulation, and resilience (Dekker, 2024; Troy et al., 2023). Across these models, disease acceptance and perceived control consistently emerge as key determinants of successful adaptation. Although these constructs are often studied independently, they represent complementary processes. This is reflected in the transactional model of stress and coping, in which perceived control primarily influences cognitive appraisal and problem-focused coping, whereas acceptance is more closely associated with emotion-focused coping (Lazarus and Folkman, 1984). Together, these perspectives suggest that successful adaptation requires both the ability to influence illness-related challenges where possible and the capacity to accept aspects of disease that cannot be controlled.

Building on these concepts, the Subjective Health Experience (SHE) model integrates acceptance and perceived control into a single framework for understanding how individuals experience and manage chronic disease (Bloem and Stalpers, 2012; Bloem et al., 2020). Rather than considering these psychological constructs separately, the SHE model combines them to classify individuals into four psychologically distinct segments that differ in health experiences, coping style, and support needs: segment 1 (high acceptance, high perceived control), segment 2 (high acceptance, low perceived control) segment 3 (low acceptance, high perceived control), and segment 4 (low acceptance, low perceived control).

Segment 4 represents the most psychologically vulnerable group, consisting of individuals who neither accept their condition nor feel capable of influencing it and who typically require intensive personal coaching and psychosocial support (Bloem and Stalpers, 2012; Bloem et al., 2020; Broekharst et al., 2023; Folkertsma et al., 2025a; Mertens et al.). They generally report the poorest subjective health and quality of life outcomes across a wide range of measures, including HRQoL, psychological wellbeing, and perceived disease burden which has been documented across chronic disease populations, including oncology (Bloem et al., 2020; Broekharst et al., 2023; Mertens et al.; Mertens et al., 2026), and now increasingly recognized in immune-mediated inflammatory diseases (Folkertsma et al., 2025a,b). This segment is also the most challenging to engage in care, as low agency and passivity make these patients less likely to seek support. Additionally, reduced trust in healthcare professionals and lower treatment adherence are linked to low perceived control and acceptance (Bloem et al., 2020; Broekharst et al., 2023; Mertens et al.; Mertens et al., 2026). Understanding the needs of this psychologically vulnerable group is therefore critical for developing targeted supportive care strategies.

Previous research applying the SHE model in patients with IMIDs has examined demographic and disease-related characteristics associated with these SHE segments (Folkertsma et al., 2026). However, recent work applying the SHE model in older cancer survivors identified substantial heterogeneity within segment 4, revealing a severe subgroup with notably worse subjective health, vitality, and frailty outcomes, independent of sociodemographic and disease-related factors (Mertens et al.). Whether similar internal differentiation exists within segment 4 among patients with IMIDs remains unknown. In particular, it is unclear whether variation within this psychologically vulnerable segment primarily reflects biomedical disease burden or psychological distress. Establishing whether such heterogeneity is present is essential to determine whether further stratification of segment 4 may be relevant for tailoring supportive care.

The present study therefore examines heterogeneity within SHE segment 4 in patients with IMIDs by subdividing this group into moderate and severe subgroups. Subjective health experience, HRQoL, psychological distress (HADS), disease activity, disease impact, and comorbidity burden will be compared between the subgroups. Based on prior evidence that psychological processes exert effects on wellbeing that are at least partially independent of perceived disease severity, we expected subgroup differences to be more strongly associated with psychological distress than with disease indicators.

## 2 Materials and methods

### 2.1 Study design and participants

This study was conducted as part of a larger project aimed at improving individualized supportive care for individuals with IMIDs by integrating the SHE model with patient-reported outcomes and clinical indicators. The study protocol was reviewed by an accredited Independent Ethics Committee and approved by the Board of Directors of Frisius MC (previously Medical Centre Leeuwarden; RTPO, nWMO 20200091, January 2021) in the Netherlands.

**FIGURE 1:**
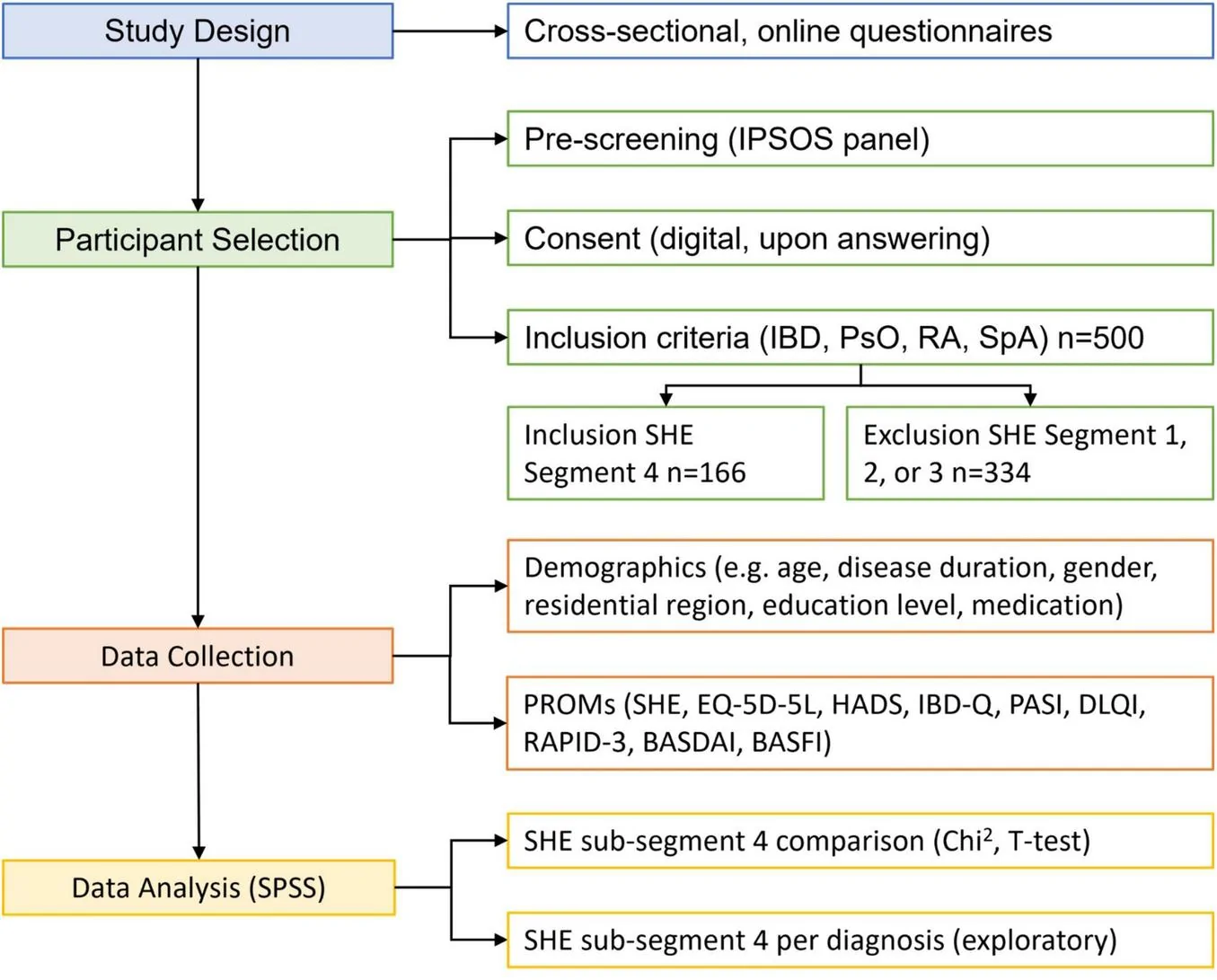
Analytical process chart. IBD, inflammatory bowel disease; PsO, psoriasis; RA, rheumatoid arthritis; axSpA, spondyloarthritis; SHE, subjective health experience; HADS, Hospital Anxiety and Depression Scale; IBDQ, Inflammatory Bowel Disease Questionnaire; DLQI, Dermatology Life Quality Index, PASI, Psoriasis Area and Severity Index; RAPID-3, Routine Assessment of Patient Index Data 3; BASDAI, Bath Ankylosing Spondylitis Disease Activity Index; BASFI, Bath Ankylosing Spondylitis Functional Index.

A cross-sectional online survey was administered by the market research firm IPSOS to a nationwide panel of Dutch adults between February and December 2023. The panel is used for a wide range of consumer and healthcare research. Panel members with a physician-confirmed diagnosis of IBD (Crohn’s disease and Ulcerative Colitis), psoriasis, rheumatoid arthritis, and axial spondyloarthritis were invited to participate. All respondents provided informed consent. Invitations were issued until the target number for each diagnostic group was reached or until IPSOS no longer had participants with that diagnosis. The final dataset included 500 participants who completed the full questionnaire, of which 166 were in segment 4. See Figure 1 for a schematic overview of the study design.

### 2.2 Measures

Questionnaires included various items on patient characteristics as well as disease-specific patient-reported outcome measures (PROMs). Demographic variables included age, gender, residential region, population density, education level, employment status, income, smoking behavior, disease duration, and comorbidity burden. Disease activity and disease impact were derived from PROMs and categorized as remission/mild, moderate, or severe to enable comparisons across diagnoses according to Folkertsma et al. (2026).

Subjective Health Experience. The SHE questionnaire was used to determine subjective health experience as well as acceptance and control (Bloem, 2012). Subjective health experience was measured using an 11-point visual analogue ladder scale ranging from 0 to 10, reflecting the average perceived health during the previous month. Participants indicated their position on the scale, where 0 represented the worst health day of the previous month and 10 represented the best health day of the previous month.

SHE model segmentation. Disease acceptance and control were each measured using three items rated on a seven-point Likert scale. Based on the scores, patients were classified into one of four SHE segments: segment 1 (high acceptance, high control), segment 2 (high acceptance, low control), segment 3 (low acceptance, high control), and segment 4 (low acceptance, low control). Cut-off values for acceptance and control were based on the median of each scale ( ≥ 4.67 and ≥ 4.33, respectively) as described by Folkertsma et al. (2026).

To explore whether clinically meaningful heterogeneity existed within the most psychologically vulnerable SHE segment, segment 4 was further stratified into moderate and severe subgroups. This exploratory stratification was based on the approach previously described by Mertens et al., who demonstrated clinically relevant heterogeneity within segment 4 using additional thresholds for acceptance and perceived control. Because previous work has shown that optimal SHE cut-offs differ between populations, with IMID-specific thresholds differing from those established in the general population, we determined subgroup thresholds within the IMID segment 4 population rather than applying the thresholds derived in older cancer survivors (Folkertsma et al., 2026). Accordingly, the median acceptance (3.33) and perceived control (3.00) scores within segment 4 were used as operational thresholds. Patients scoring at or below both medians were classified as the severe subgroup, whereas those scoring above the median on at least one dimension were classified as the moderate subgroup. This theory-driven exploratory approach preserved the conceptual framework of the SHE model while allowing investigation of whether clinically meaningful heterogeneity exists within the predefined vulnerable segment. Health-related quality of life (HRQoL). General health-related quality of life was assessed using the EQ-5D-5L questionnaire (EuroQol Group, 1990; Greiner et al., 2003). The instrument measures five dimensions of health status: mobility, self-care, usual activities, pain/discomfort, and anxiety/depression. Each dimension was rated on a five-level scale ranging from no problems to extreme problems. Dimension scores were summed to produce a total score ranging from 5 to 25, with higher scores indicating poorer health-related quality of life.

Psychological distress. Psychological distress was assessed using the Hospital Anxiety and Depression Scale (HADS) (Zigmond and Snaith, 1983). The HADS consists of two 7-item subscales measuring anxiety (HADS-A) and depression (HADS-D). Items are rated on a 4-point scale (0–3), yielding subscale scores of 0–21 and a total distress score of 0 to 42, with higher scores indicating greater symptom severity. The instrument was developed for use in medical populations and minimizes confounding by somatic symptoms.

Disease activity and impact. To facilitate cross-disease comparison, specific items were selected from disease-specific PROMs to form disease activity and disease impact, as described by Folkertsma et al. (2026). Disease activity refers to the clinical symptoms associated with the disease, such as pain, stiffness, itching, and bloating, which reflect the current severity of the disease. In contrast, disease impact encompasses measures of disease-related quality of life and functional outcomes, reflecting how the disease affects a patient’s daily functioning and overall well-being. The selection of measures and items was established through group discussions with three physicians with expertise in one or more of the included IMIDs to ensure conceptual comparability across diseases. Scores were calculated according to the respective instrument guidelines, adjusted where only selected items were used, and categorised as remission/mild, moderate, or severe to enable comparison across diseases. The specific measures, selected items, scoring procedures, cut-offs, and supporting literature are provided in Supplementary Table S1. For IBD, the Inflammatory Bowel Disease Questionnaire (IBDQ) (Guyatt et al., 1989) was used; for PsO, the Dermatology Life Quality Index (DLQI) (Finlay and Khan, 1994) and Psoriasis Area and Severity Index (PASI) (Fredriksson and Pettersson, 1978); for RA, the Routine Assessment of Patient Index Data 3 (RAPID-3) (Pincus et al., 2008); and for axSpA, the Bath Ankylosing Spondylitis Disease Activity Index (BASDAI) (Garrett et al., 1994) and Bath Ankylosing Spondylitis Functional Index (BASFI) (Calin et al., 1994).

### 2.3 Statistical analysis

Patient characteristics were summarized using descriptive statistics. Assumptions for parametric testing were evaluated; continuous variables were inspected for normality and homogeneity of variance and were deemed acceptable for analysis. Differences between the moderate and severe subgroups within segment 4 were examined using independent-samples *t*-tests for continuous variables and Pearson’s χ^2^ tests for categorical variables. To examine whether differences between the moderate and severe subgroups remained after adjustment for demographic and clinical characteristics, separate multiple linear regression analyses were performed for SHE, HRQoL, HADS anxiety, HADS depression, and HADS total. Models were adjusted for age, gender, education level, diagnosis, and comorbidity burden. To assess whether subgroup differences were consistent across diagnoses, an exploratory analysis was done for IBD, PsO, RA, and axSpA. No statistical tests were performed for this analysis due to limited subgroup size. All analyses were performed using SPSS (version 28). Statistical significance was set at *p* < 0.05.

## 3 Results

### 3.1 Sub-segmentation of segment 4

Within segment 4, 96 patients (57.8%) were classified in the moderate subgroup and 70 patients (42.2%) in the severe subgroup. The characteristics of both subgroups are presented in Table 1. No significant differences were observed for age, employment status, or smoking behavior. Lower educational attainment was more common in the moderate subgroup. The distribution of comorbidities did not differ significantly between subgroups, nor did the mean number of comorbidities.

**TABLE 1:** Patient characteristics of moderate and severe subgroups within SHE segment 4.

Variable	Moderate segment 4	Severe segment 4	T/ χ^2^	*p*-value
*N*	96	70		
Age in y	57.9 ± 12.3	61.3 ± 11.7	T = −1.82	0.071
Disease duration in y	18.7 ± 13.8	19.2 ± 16.2	T = −0.22	0.830
Gender			χ^2^ = 0.58	0.632
Female	61.5%	57.1%		
Male	38.5%	42.9%		
Residential region			χ^2^ = 1.34	0.511
North and East NL	31.3%	34.3%		
West NL	50.0%	41.4%		
South NL	18.8%	24.3%		
Area population density			χ^2^ = 0.42	0.810
< 200 inhab/km^2^	15.6%	12.9%		
200–999 inhab/km^2^	37.5%	51.4%		
≥ 1000 inhab/km^2^	46.9%	35.7%		
Education level			χ^2^ = 7.08	0.029
Low	38.5%	20.3%		
Middle	37.5%	42.0%		
High	24.0%	37.7%		
Income			χ^2^ = 2.95	0.228
< €36.500	63.3%	52.7%		
36.500–43.500	13.9%	10.9%		
> 43.500	22.8%	36.4%		
Employment status			χ^2^ = 0.38	0.829
Employed	31.3%	28.6%		
Unemployed	37.5%	35.7%		
Inactive	31.3%	35.7%		
Smoking			χ^2^ = 2.64	0.267
Never	28.1%	34.3%		
Ex-smoker	45.8%	50.0%		
Current smoker	26.0%	15.7%		
Comorbidity burden^a^			χ^2^ = 5.10	0.164
None	46.9%	51.4%		
1	40.6%	32.9%		
2	5.2%	12.9%		
3	7.3%	2.9%		
Comorbidities^a^	0.73 ± 0.86	0.67 ± 0.81	T = 0.44	0.663
SHE	5.30 ± 1.48	3.99 ± 1.70	T = 5.32	< 0.001
HRQoL	10.55 ± 3.17	13.09 ± 3.89	T = −4.62	< 0.001
HADS				
Anxiety	7.13 ± 4.56	8.70 ± 4.40	T = −2.22	0.028
Depression	7.38 ± 3.99	10.15 ± 4.65	T = −4.06	< 0.001
Total	14.5 ± 7.48	18.74 ± 8.04	T = −3.43	< 0.001
Disease activity			χ^2^ = 5.07	0.079
Remission/mild	46.7%	40.6%		
Moderate	32.6%	23.2%		
Severe	20.7%	36.2%		
Disease impact			χ^2^ = 5.50	0.064
Remission/mild	43.9%	29.2%		
Moderate	30.5%	27.7%		
Severe	25.6%	43.1%		

^a^Comorbidities included self-reported physician-confirmed diagnoses of a second IMID (IBD, PsO, RA, or axSpA), chronic obstructive pulmonary disease, asthma, or type 1 or type 2 diabetes mellitus.

**FIGURE 2:**
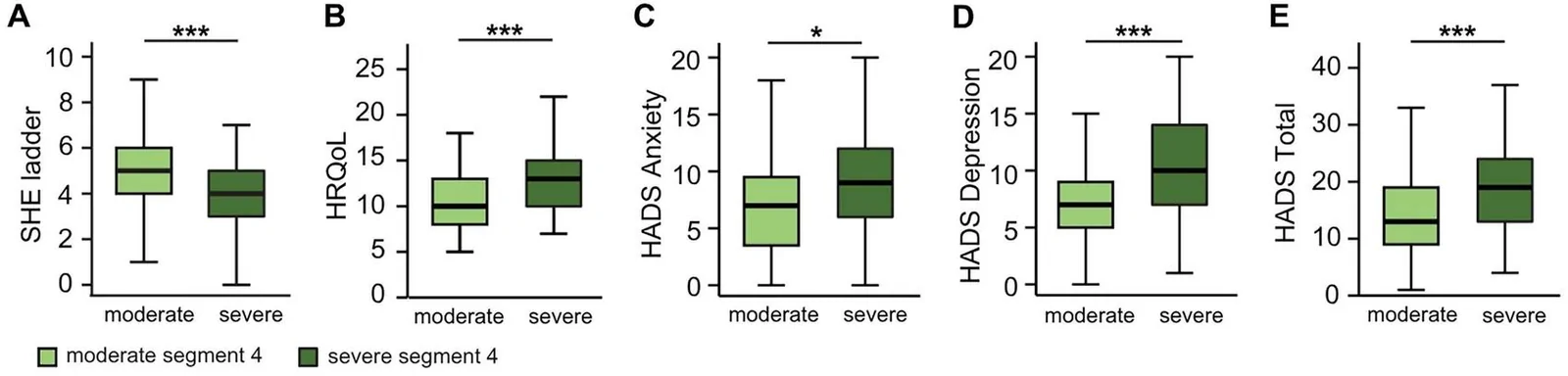
Boxplots of patient-reported outcomes for the moderate and severe subgroups within SHE segment 4. Panels display **(A)** SHE ladder, **(B)** HRQoL, **(C)** HADS anxiety, **(D)** HADS depression, and **(E)** HADS total score. Boxes represent the interquartile range (IQR) with the median indicated by the horizontal line. Higher HRQoL scores reflect poorer quality of life; higher SHE scores reflect better experienced health; higher HADS scores reflect greater psychological distress. Statistical significance is indicated as follows: *p* < 0.05 (*) and *p* < 0.001 (***).

Subjective health and HRQoL were significantly lower in the severe subgroup compared with the moderate subgroup, while symptoms of anxiety, depression, and overall psychological distress were significantly higher (Figure 2 and Table 2). Patient-reported disease activity did not differ significantly between the moderate and severe subgroups, nor did patient-reported disease impact, although severe disease activity and impact were more common in the severe subgroup.

**TABLE 2:** Adjusted associations between severe subgroup membership and patient-reported outcomes.

Outcome	B^a^	β	95% CI	*p*-value	Adjusted R^2^
**SHE**	−1.42	−0.413	−1.95 to −0.90	< 0.001	0.142
**HRQoL**	2.45	0.329	1.35 to 3.55	< 0.001	0.196
HADS					
Anxiety	2.04	0.221	0.58 to 3.50	0.006	0.088
Depression	2.79	0.307	1.33 to 4.25	< 0.001	0.081
Total	4.71	0.291	2.09 to 7.33	< 0.001	0.076

^a^B coefficients represent the adjusted difference between the severe and moderate segment 4 (reference group). Models were adjusted for age, gender, education, diagnosis, and comorbidity burden.

### 3.2 Adjusted analyses of subgroup differences

To determine whether subgroup differences remained after adjustment for demographic and clinical characteristics, separate multiple linear regression analyses were performed for SHE, HRQoL, HADS anxiety, HADS depression, and HADS total (Table 2).

After adjustment for age, gender, education, diagnosis, and comorbidity burden, membership of the severe subgroup remained significantly associated with lower SHE and worse HRQoL, anxiety, depression, and total psychological distress (all *p* ≤ 0.006). Among the covariates, only greater comorbidity burden was independently associated with worse HRQoL (*B* = 1.03, 95% CI 0.39 to 1.67, β = 0.235, *p* = 0.002), whereas older age was associated with lower anxiety scores (*B* = −1.22, 95% CI −2.21 to −0.22, β = −0.197, *p* = 0.017). No other demographic or clinical characteristics were significantly associated with the outcomes.

### 3.3 Subgroup differences within SHE segment 4 across diagnoses

Exploratory analyses of moderate and severe subgroups within segment 4, stratified by diagnosis, indicated differences in both subgroup distribution and outcome patterns. Among patients with IBD, PsO, and axSpA, most were classified as moderate (64–70%), whereas in RA most patients were classified as severe (58%). Sample sizes per diagnosis were modest (IBD: *n* = 34; PsO: *n* = 53; RA: *n* = 59; axSpA: *n* = 20). Across all diagnoses, the severe subgroup reported poorer subjective health, lower HRQoL, and higher levels of psychological distress compared with the moderate subgroup. These differences appeared largest in IBD, smaller in PsO, and least pronounced in RA and axSpA. In contrast, disease activity and disease impact were largely comparable between subgroups across diagnoses.

## 4 Discussion

This study explored heterogeneity within the most psychologically vulnerable subgroup of the Subjective Health Experience model among patients with IMIDs. Within segment 4, characterized by low acceptance and low perceived control, we identified a moderate and a severe subgroup. Patients in the severe subgroup reported worse subjective health experience, lower HRQoL, and higher psychological distress (anxiety and depression), while disease activity, disease impact, and comorbidity burden did not differ between groups. These results indicate that within the most vulnerable SHE segment, subgroup differentiation was primarily associated with psychological and emotional wellbeing, rather than perceived disease burden. Importantly, these associations remained after adjustment for age, gender, education, diagnosis, and comorbidity burden, indicating that the observed differences cannot be attributed to these demographic and clinical characteristics.

It should be recognized that the moderate and severe subgroups were defined using acceptance and perceived control, psychological constructs that are conceptually related to subjective health and psychological distress. Consequently, some degree of association with SHE and HADS is expected. However, the outcomes examined in this study represent broader dimensions of health experience and emotional functioning rather than the defining constructs themselves. The observation that subgroup differences extended consistently across subjective health, HRQoL, anxiety, and depressive symptoms, and remained after adjustment for demographic and clinical characteristics, supports the interpretation that variation in acceptance and perceived control is associated with clinically meaningful differences in overall psychological functioning rather than merely the operational subgroup definition. Nevertheless, because the subgroup classification was itself based on acceptance and perceived control, these findings should be interpreted as supporting, rather than definitively establishing, the clinical validity of this distinction.

Patient-reported indicators of disease status were not associated with the observed heterogeneity within segment 4. Disease activity and disease impact showed no significant differences between moderate and severe subgroups despite clear differences in subjective health and psychological distress. Comorbidity burden was comparable, consistent with evidence that multimorbidity explains only part of subjective wellbeing variance (Fortin et al., 2006; Nicholson et al., 2019). This is consistent with findings from IMID research showing that patient-reported disease measures correlate only moderately with subjective health outcome (Folkertsma et al., 2026; Matcham et al., 2016; Mikocka-Walus et al., 2016). Together, these findings suggest that subjective health experience and depressive symptom severity capture a vulnerability dimension that is largely independent of perceived disease burden.

Psychological distress emerged as a central differentiating feature of the severe segment 4 profile. Patients in the severe subgroup had higher levels of anxiety, depressive symptoms, and total psychological distress on the HADS, with the largest differences observed for depressive symptoms. The severe subgroup’s depression scores suggest the possible presence of a mood disorder (for IBD even being in the clinically relevant range; ≥ 11) (Zigmond and Snaith, 1983). Notably, scores in the moderate subgroup were not in a healthy range either, indicating that neither subgroup can be considered psychologically well-functioning. The elevated distress observed in the moderate subgroup, combined with considerable score variability within this group, may indicate that a subset of these patients experiences considerable psychological burden despite not belonging to the most severe subgroup. This finding highlights the potential value of early psychological assessment within segment 4. Whether the moderate subgroup represents a transitional profile associated with increasing psychological distress over time cannot be determined from the present cross-sectional data and requires longitudinal investigation.

In the SHE model, segment 4 is characterized by passivity and inertia, reflecting the helplessness and reduced sense of control described in models of depression (Abramson et al., 1989; Bandura, 1977). Low self-efficacy and control beliefs are well-established vulnerability indicators for depression (Bandura, 1977), particularly in chronic disease where poor adjustment and low acceptance can reinforce depressive cognitions (Helgeson and Zajdel, 2017). Both depressive symptoms and anxiety were significantly higher in the severe subgroup, with depression showing a substantially larger effect. While anxiety was also elevated, the stronger association with depression suggests that the severe segment 4 profile is more closely aligned with depressive processes such as anhedonia and behavioral withdrawal, than the hypervigilance typically associated with anxiety disorders. This finding emphasizes the importance of screening for depressive symptoms specifically in this subgroup.

The diagnosis-specific analyses should be interpreted as exploratory because subgroup sizes within individual IMIDs were modest. Nevertheless, poorer subjective health, HRQoL, and psychological outcomes were consistently observed in the severe subgroup across all diagnoses, suggesting that the distinction between moderate and severe vulnerability may be relevant across different IMIDs. These patterns are in line with previous research demonstrating an important role for psychological factors across IMIDs (Knowles et al., 2018; Mikocka-Walus et al., 2019; Peppas et al., 2021). Numerically larger subgroup differences were observed in patients with IBD, although these observations should be interpreted cautiously given the limited statistical power. Previous studies have similarly reported substantial psychological burden in patients with IBD, supporting the need for further investigation in larger cohorts (Broekharst et al., 2023). Numerically smaller subgroup differences were observed in RA. Whether this reflects a more homogeneous distribution of psychological vulnerability or is attributable to sampling variation should be investigated in larger studies.

These findings raise important questions for clinical practice. If future longitudinal research confirms that psychological vulnerability represents a key determinant of poor health experiences beyond disease burden, standard pharmacological treatment is unlikely to address the principal source of distress in this subgroup. An important consideration is how the SHE model relates to established screening instruments such as the HADS. The HADS identifies symptoms of anxiety and depression, whereas the SHE model assesses disease acceptance and perceived control. Rather than replacing established screening instruments, the SHE model provides complementary information that may help guide personalized communication and supportive care, as demonstrated in our previous qualitative study (Folkertsma et al., 2025b). Such an approach may be particularly valuable given that psychological support within specialty IMID care remains inconsistent and underutilized. For example, a survey of IBD patients found that only 15.2% were currently seeing a mental health practitioner, only 16.1% had been asked about their mental health by their IBD specialist, and nearly 68% of those with severe psychological vulnerability were not receiving support (Mikocka-Walus et al., 2019). In this context, the SHE model offers a structured framework for identifying patients who may benefit from more intensive psychosocial assessment and supportive care. Within the this framework, patients in segment 4 are considered likely to benefit from more intensive psychosocial assessment and may require specialist support beyond that provided by the treating healthcare professional. Such support is intended to facilitate incremental improvements in acceptance and perceived control (Mertens et al.). However, whether SHE-guided supportive care translates into improved patient outcomes remains to be established in prospective intervention studies. The challenge also extends beyond identifying vulnerable patients. Segment 4 patients are characterized by disengagement and reduced agency, rendering them less likely to seek psychological support or proactively navigate existing care pathways independently (Mertens et al.). Consequently, care systems that rely primarily on patient initiative may systematically fail those with the greatest need. The clinical utility of the SHE model therefore depends not only on identifying vulnerable patients, but also on whether healthcare systems are structured to act on this information.

This study has several limitations. First, the cross-sectional design precludes causal inference. Consequently, the temporal and directional relationships between depressive symptoms, perceived control, acceptance, and subjective health cannot be determined. Although the observed associations are consistent with existing theoretical models and previous research, longitudinal studies are required to establish the direction of these relationships. Second, disease activity and disease impact were derived from disease-specific patient-reported questionnaires rather than clinician-rated assessments or objective indicators of inflammation. Consequently, these measures may not fully reflect biological disease activity. Third, modest subgroup sizes within individual diagnoses limited statistical power for diagnosis-specific analyses. Fourth, the subdivision of segment 4 was based on operational thresholds derived from the distribution of acceptance and perceived control within the IMID cohort. Although this approach is consistent with the theory-driven and population-specific segmentation strategy of the SHE model, the resulting subgroup classification remains exploratory and requires validation in independent IMID cohorts. Alternative approaches, such as latent profile analysis or cluster analysis, may identify different subgroup structures. Future studies should validate the proposed thresholds in independent IMID cohorts and compare them with data-driven classification methods to determine the most robust approach for identifying clinically meaningful heterogeneity within segment 4. Finally, information on previous or current mental health diagnoses, psychological treatment, and psychotropic medication use was unavailable. As these factors may influence psychological functioning and adaptation to chronic disease, residual confounding cannot be excluded.

Future research should build on these findings in several directions. Longitudinal studies are needed to examine the stability of segment 4 profiles over time and to clarify potential causal relationships between depressive symptoms, perceived control, acceptance, and subjective health outcomes. In addition, intervention and causal modelling studies could evaluate whether targeting depressive symptoms, perceived control, and acceptance improves subjective health experience within psychologically vulnerable subgroups. Finally, given the exploratory indications of variation across IMID diagnoses, future research should examine whether the relative contribution of acceptance and perceived control differs systematically between conditions and how this may inform more tailored supportive care strategies.

## 5 Conclusion

Overall, this study extends prior work on the SHE model in IMIDs by demonstrating that meaningful psychological heterogeneity exists within the most vulnerable segment. Differences between moderate and severe segment 4 subgroups were more strongly associated with psychological wellbeing than with disease activity, disease impact, or comorbidity burden. These findings suggest that psychologically vulnerable patients with IMIDs do not constitute a uniform group, and that further differentiation within segment 4 may have clinical relevance. The SHE model may help distinguish between patients who share a similar level of disease burden but differ substantially in psychological vulnerability, thereby providing a structured basis for more personalized supportive care.

## Data Availability

The data supporting the findings of this study are available from the corresponding author upon reasonable request, subject to applicable privacy and data protection requirements.
