# Supplementary material for: Psychological distress differentiates subjective health within a vulnerable subgroup of patients with immune-mediated inflammatory diseases
Source: Front Psychol. 2026 Aug 31;17:1884317. doi: 10.3389/fpsyg.2026.1884317 (PMC13572688; doi:10.3389/fpsyg.2026.1884317)
Supplement: Supplementary file 1 [file Table_1.docx]

Disease activity and disease impact proxy variables were derived from disease-specific measures, using either complete instruments or selected items or domains, depending on the disease and construct. Questionnaires were administered using validated Dutch-language versions. English item wording is presented for readability. The full questionnaires are copyrighted and available from the respective copyright holders; only the analysed items are presented here for transparency. Table S1 presents the operationalisation, scoring, and categorisation of the proxy variables, followed by the specific items included in each proxy.

Table S1: Disease activity and disease impact proxy categorisation and rationale

| Diagnosis | Construct | Measure | Included items, section or domains | Original → adjusted score | Final categorisation | Basis |  |
| --- | --- | --- | --- | --- | --- | --- | --- |
| IBD | Disease activity | IBDQ | Domain: Bowel. Assessing pain, obstipation, stool, and gas | Original: 32-224  Adjusted: 10-70 | **Category** \| **Range 10-70**; Remission/mild \| >47; Moderate \| 38-47; Severe \| ≤37 | (1) |  |
| IBD | Disease impact | IBDQ | Domains: systemic, emotional and social, captures both functional limitations and quality of life impairments due to IBD. Item 2 (*feeling tired or worn out*) was excluded. | Original: 32-224  Adjusted: 21-147 | **Category** \| **Range 21-147**; Remission/mild \| > 98; Moderate \| 79-98; Severe \| ≤ 79 | (1) |  |
| PsO | Disease activity | PASI | Complete questionnaire assessing percentage of body that is affected by the disease | Original: 0-72  Adjusted: 0-72 | **Category** \| **Range**; Remission/mild \| ≤6.9; Moderate \| 7.0-12.0; Severe \| >12.0 | (2) |  |
| PsO | Disease impact | DLQI | Questionnaire excluding the first question (H*ow itchy, sore, painful or stinging has your skin been?*). It assesses, work, social relationships, and self-image. | Original: 0-30  Adjusted: 0-27 | **Category** \| **Range 0-27**; Remission/mild \| ≤5.39; Moderate \| 5.40-9.00; Severe \| >9.00 | (3, 4) |  |
| RA | Disease activity | RAPID-3 | Section 3: Self-rated painful joint count | Original: 0-48  Adjusted: 0-48 | **Category** \| **Range**; Remission/mild \| ≤9; Moderate \| 10-16; Severe \| >16 | (5) |  |
| RA | Disease impact | RAPID-3 | Section 1: Physical abilities  Section 4: Personal health assessment | Original: 0-30  Adjusted: 0-20 | **Category** \| **Range 0-20**; Remission/mild \| ≤4.0; Moderate \| 4.01-8.0; Severe \| >8.0 | (6, 7) |  |
| axSpA | Disease activity | BASDAI | Questionnaire except item 1 (H*ow tired are you?*) assessing spinal pain, peripheral joint pain, and morning stiffness. | Original: 0-10  Adjusted: 0-9 | **Category** \| **Range 0-9**; Remission/mild \| ≤1.8; Moderate \| 1.9-5.4; Severe \| >5.4 | (8, 9) |  |
| axSpA | Disease impact | BASFI | Measures physical function and the ability to perform daily activities affected by axial spondyloarthritis. | Original: 0-10  Adjusted: 0-10 | **Category** \| **Range 0-10**; Remission/mild \| ≤3; Moderate \| 3.1-7; Severe \| >7 | (8) |  |

- Where selected items or domains were used, score ranges and categorisation thresholds were proportionally adjusted according to the number of included items.

Exact analysed items:

**IBD – Disease activity (IBDQ)**
Items analysed:

1. How frequent have your bowel movements been during the last 2 weeks?
2. How much of the time during the last 2 weeks have your bowel movements been loose?
3. How often during the last 2 weeks have you been troubled by cramps in your abdomen?
4. How often during the last 2 weeks have you been troubled by pain in the abdomen?
5. Overall, in the last 2 weeks how much of a problem have you had with passing large amounts of gas?
6. How much of the time during the last 2 weeks have you been troubled by a feeling of abdominal bloating?
7. How much of the time during the last 2 weeks have you had a problem with rectal bleeding with your bowel movements?
8. How much of the time during the last 2 weeks have you been troubled by a feeling of having to go to the bathroom even though your bowels were empty?
9. How much of the time during the last 2 weeks have you been troubled by accidental soiling of your underpants?
10. How much of the time during the last 2 weeks have you been troubled by nausea or feeling sick to your stomach?

**IBD – Disease impact (IBDQ)**
Items analysed:

1. How often during the last 2 weeks have you felt frustrated, impatient, or restless?
2. How often during the last 2 weeks have you been unable to attend school or do your work because of your bowel problem?
3. How much energy have you had during the last 2 weeks?
4. How often during the last 2 weeks did you feel worried about the possibility of needing to have surgery because of your bowel problem?
5. How often during the last 2 weeks have you had to delay or cancel a social engagement because of your bowel problem?
6. How often during the last 2 weeks have you felt generally unwell?
7. How often during the last 2 weeks have you been troubled because of fear of not finding a washroom?
8. How much difficulty have you had, as a result of your bowel problems, doing leisure or sports activities you would have liked to have done during the last 2 weeks?
9. How often during the last 2 weeks have you had problems getting a good night’s sleep, or been troubled by waking up during the night?
10. How often during the last 2 weeks have you felt depressed or discouraged?
11. How often during the last 2 weeks have you had to avoid attending events where there was no washroom close at hand?
12. Overall, in the last 2 weeks, how much of a problem have you had maintaining or getting to, the weight you would like to be at?
13. Many patients with bowel problems often have worries and anxieties related to their illness. These include worries about getting cancer, worries about never feeling any better and worries about having a relapse. In general, how often during the last 2 weeks have you felt worried or anxious?
14. How often during the last 2 weeks have you felt relaxed and free of tension?
15. How much of the time during the last 2 weeks have you felt embarrassed as a result of your bowel problem?
16. How much of the time during the last 2 weeks have you felt tearful or upset?
17. How much of the time during the last 2 weeks have you felt angry as a result of your bowel problem?
18. To what extent has your bowel problem limited sexual activity during the last 2 weeks?
19. How much of the time during the last 2 weeks have you felt irritable?
20. How often during the past 2 weeks have you felt a lack of understanding from others?
21. How satisfied, happy, or pleased have you been with your personal life during the past 2 weeks?

**PsO – Disease activity (PASI)**
Items analysed: all

**PsO – Disease impact (DLQI)**
Items analysed:

1. Over the last week, how embarrassed or self-conscious have you been because of your skin?
2. Over the last week, how much has your skin interfered with you going shopping or looking after your home or garden?
3. Over the last week, how much has your skin influenced the clothes you wear?
4. Over the last week, how much has your skin affected any social or leisure activities?
5. Over the last week, how much has your skin made it difficult for you to do any sport?
6. Over the last week, has your skin prevented you from working or studying? If "No", over the last week how much has your skin been a problem at work or studying?
7. Over the last week, how much has your skin created problems with your partner or any of your close friends or relatives?
8. Over the last week, how much has your skin caused any sexual difficulties?
9. Over the last week, how much of a problem has the treatment for your skin been, for example by making your home messy, or by taking up time?

**RA – Disease activity (RAPID-3/MDHAQ)**
Items analysed:

Select the amount of pain you have in each joint:

1. Finger Left
2. Wrist Left
3. Elbow Left
4. Shoulders Left
5. Hip Left
6. Knee Left
7. Ankle Left
8. Toe Left
9. Finger Right
10. Wrist Right
11. Elbow Right
12. Shoulder Right
13. Hip Right
14. Knee Right
15. Ankle Right
16. Toe Right

**RA – Disease impact (RAPID-3/MDHAQ)**
Items analysed:

1. Dress yourself, including shoelaces and buttons?
2. Get in and out of bed?
3. Lift a full cup or glass to your mouth?
4. Walk outdoors on flat ground?
5. Wash and dry your body?
6. Bend down to pick up clothing from the floor?
7. Turn faucets on and off?
8. Get in and out of a car?
9. Walk two miles or three kilometers, if you wish?
10. Participate in recreational activities and sports as you would like, if you wish?
11. Considering all the ways in which illness and health conditions may affect you at this time, please indicate below how you are doing: 0-10

**axSpA – Disease activity (BASDAI)**
Items analysed:

1. How would you describe the overall level of pain / swelling in the neck, back or hip you have had ?
2. How would you describe the overall level of pain / swelling in joints other than neck, back or hips you have had?
3. How would you describe the overall level of discomfort you have had from any areas tender to touch or pressure?
4. How would you describe the overall level of morning stiffness you have had from the time you wake up?
5. How long did your morning stiffness last from the time you wake up?

**axSpA – Disease impact (BASFI)**
Items analysed: all

1.	Sandborn WJ, D'Haens GR, Reinisch W, Panés J, Chan D, Gonzalez S, et al. Guselkumab for the Treatment of Crohn's Disease: Induction Results From the Phase 2 GALAXI-1 Study. Gastroenterology. 2022;162(6):1650–64.e8.

2.	Schmitt J, Wozel G. The Psoriasis Area and Severity Index Is the Adequate Criterion to Define Severity in Chronic Plaque-Type Psoriasis. Dermatology. 2005;210(3):194–9.

3.	Basra MK, Fenech R, Gatt RM, Salek MS, Finlay AY. The Dermatology Life Quality Index 1994-2007: a comprehensive review of validation data and clinical results. Br J Dermatol. 2008;159(5):997–1035.

4.	Manchanda Y, De A, Das S, Chakraborty D. Disease Assessment in Psoriasis. Indian Journal of Dermatology. 2023;68(3).

5.	Castrejón I, Yazici Y, Pincus T. Patient self-report RADAI (Rheumatoid Arthritis Disease Activity Index) joint counts on an MDHAQ (Multidimensional Health Assessment Questionnaire) in usual care of consecutive patients with rheumatic diseases other than rheumatoid arthritis. Arthritis Care & Research. 2013;65(2):288–93.

6.	Pincus T, Swearingen CJ, Bergman MJ, Colglazier CL, Kaell AT, Kunath AM, et al. RAPID3 (Routine Assessment of Patient Index Data) on an MDHAQ (Multidimensional Health Assessment Questionnaire): Agreement with DAS28 (Disease Activity Score) and CDAI (Clinical Disease Activity Index) activity categories, scored in five versus more than ninety seconds. Arthritis Care & Research. 2010;62(2):181–9.

7.	Pincus T, Swearingen CJ, Bergman M, Yazici Y. RAPID3 (Routine Assessment of Patient Index Data 3), a rheumatoid arthritis index without formal joint counts for routine care: proposed severity categories compared to disease activity score and clinical disease activity index categories. J Rheumatol. 2008;35(11):2136–47.

8.	Wariaghli G, Allali F Fau - Berrada K, Berrada K Fau - Idrissi Z, Idrissi Z Fau - Hmamouchi I, Hmamouchi I Fau - Abouqal R, Abouqal R Fau - Hajjaj-Hassouni N, et al. Normative values for the Bath Ankylosing Spondylitis Functional Index in the general population compared with ankylosing spondylitis patients in Morocco. (1471-2474 (Electronic)).

9.	Georgiadis S OL, Michelsen B, Kvien T, Rasmussen S, Závada J, Bubová K, Glintborg B, Loft A, Rodrigues A, Santos M, Ciurea A, Nissen M, Kuusalo L, Rutanen J, Rotar Z, Perdan-Pikmajer K, Gudbjornsson B, Palsson O, DiGuiseppe D, Ostergaard M, Hetland M. Defining BASDAI Cut-offs for Disease Activity States in Axial Spondylarthritis – Results from the EuroSpA Collaboration [abstract]. Arthritis Rheumatol2024.
